# Molecular Events of the Crossbridge Cycle Reflected in the Force–Velocity Relationship of Activated Muscle

**DOI:** 10.3389/fphys.2022.846284

**Published:** 2022-03-10

**Authors:** Kathryn N. Seow, Chun Y. Seow

**Affiliations:** ^1^Faculty of Land and Food Systems, University of British Columbia, Vancouver, BC, Canada; ^2^Department of Pathology and Laboratory Medicine, University of British Columbia, Vancouver, BC, Canada; ^3^Centre for Heart Lung Innovation, Providence Health Care/St. Paul’s Hospital, University of British Columbia, Vancouver, BC, Canada

**Keywords:** muscle mechanics, isometric contraction, isotonic shortening, power output, internal load, crossbridge cycle

## Abstract

Muscles convert chemical energy to mechanical work. Mechanical performance of a muscle is often assessed by the muscle’s ability to shorten and generate power over a range of loads or forces, characterized by the force–velocity and force–power relationships. The hyperbolic force–velocity relationship of muscle, for a long time, has been regarded as a pure empirical description of the force–velocity data. Connections between mechanical manifestation in terms of force–velocity properties and the kinetics of the crossbridge cycle have only been established recently. In this review, we describe how the model of Huxley’s crossbridge kinetics can be transformed to the hyperbolic Hill equation, and link the changes in force–velocity properties to molecular events within the crossbridge cycle driven by ATP hydrolysis. This allows us to reinterpret some findings from previous studies on experimental interventions that altered the force–velocity relationship and gain further insight into the molecular mechanisms of muscle contraction under physiological and pathophysiological conditions.

## Introduction

An activated muscle is able to exert force or carry a load while shortening. The velocity of shortening decreases as the load or force on the muscle increases. The force–velocity relationship is not linear, rather, it can be described by a hyperbolic function, first proposed by Hill (1938):


(1) (F+a)(V+b)=c

where F and V are muscle force and velocity, respectively, and *a*, *b*, and *c* are Hill’s constants. Data from Hill’s (1938) study suggested that the mechanics of muscle contraction could be linked to the muscle’s energy metabolism, because the same hyperbolic force–velocity relationship could be derived from heat measurements and the constant *a* was derived from the thermal constant of shortening heat—α (Hill, 1938). A later study by Hill (1964) found that α was not a constant, but a function of shortening velocity and load, therefore α is not equivalent to *a*. The Hill equation has since been used as an empirical mathematical description of the force–velocity data (Abbott and Wilkie, 1953), until recently (Seow, 2013).

Our current understanding of the characteristic force–velocity behavior of muscle is based on Huxley’s (1957) kinetic model of cyclic interaction between myosin crossbridges and actin filaments. From a molecular basis, Huxley has demonstrated that the force–velocity relationship does not originate from the behavior of individual crossbridges, but from the collective action of all activated crossbridges in the muscle as they go through, asynchronously, the energy-dependent cyclic interaction with actin filaments. It is known that Huxley’s kinetic model predicts a force–velocity relationship that can be well-fitted by the Hill equation. It has been shown that this is not a coincidence, but rather that the two forms of description are mathematically identical when certain conditions are met (Seow, 2013). The recognition allowed us to link the molecular mechanisms of crossbridge cycle to the emergent relationship between muscle force and shortening velocity. Changes in the curvature of the force–velocity curve, for example, can now be interpreted in terms of changes in the rates of crossbridge attachment to, and detachment from, the actin filaments. Because the force–power relationship of a muscle can be derived from its force–velocity relationship, muscle performance in terms of power output can be understood at the crossbridge level through the changes in the force–velocity properties.

## Force–Velocity Relationship: How is it Assessed?

### Acquisition and Assessment of Force–Velocity Data

**Figure 1:**
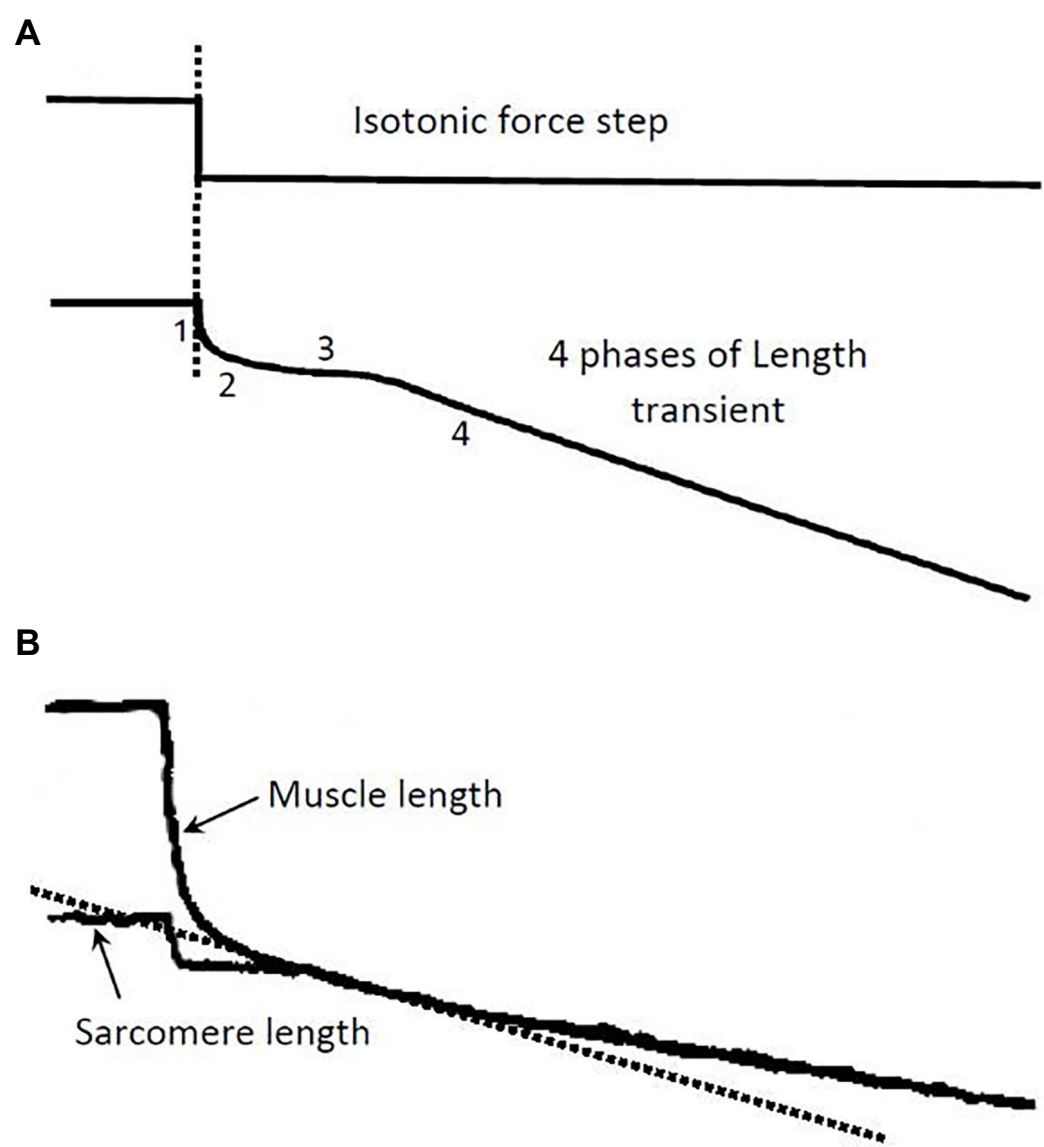
**(A)** Illustration of a step release in force and the ensuing length transient. The 4 phases of the length response and their underlying causes are described in the text. The isotonic load after the release is kept constant during the transient and the phase 4 contraction (Modified from Seow, 2013). **(B)** Records from an experiment showing length response to a force step. A skinned single fiber of rabbit psoas muscle was used in the experiment. Both the fiber length and the laser-diffracted sarcomere length signals were recorded. The initial slope (dotted line) of the phase 4 response in the sarcomere length record is taken as the shortening velocity corresponding to the isotonic load.

In isolated muscle preparations, force–velocity data are usually obtained using a method called isotonic quick release. Typically, during an isometric contraction, a step (or isotonic) release is applied so that the muscle shortens under a constant load, as illustrated in Figure 1A. It is known that the length change in response to the force step consists of four phases (Civan and Podolsky, 1966) and that each can be traced to certain cellular and subcellular origins (Ford et al., 1977). Phase 1 is primarily an elastic recoil immediately following the step release of tension. In striated muscle preparations with little stray compliance, phase 1 response stems mainly from the crossbridge and actin filament elasticity. Muscle stiffness assessed from the phase 1 response can therefore be used as an approximate index of the number of attached crossbridges. The phase 2 response originates mainly from the synchronous power strokes of some crossbridges after the step release (Dobbie et al., 1998; Suzuki et al., 1998). Phase 3 occurs when some of the crossbridges reach the end of their power stroke, and others start to detach from actin filaments. Phases 1 to 3 are transient responses, unlike phase 4, which occurs when the crossbridges enter steady-state asynchronous cycles. In force–velocity measurements, the initial slope of the phase 4 response is taken as the steady-state shortening velocity of the muscle under the externally applied isotonic load. The force–velocity properties of the muscle are therefore steady-state properties.

Many muscle preparations used in experiments contain a substantial amount of stray compliance, which usually stems from the crushed ends of the muscle preparation. The stray compliance is viscoelastic in nature (Seow and Ford, 1992), and its recoil during the step release can obscure the transient response of the muscle. Figure 1B shows a length response from a skinned (membrane-permeabilized) rabbit psoas fiber (cell), and a sarcomere length response from the same fiber. The muscle length response contains stray compliance which obscures the transients, whereas the sarcomere length response is free of the influence of the stray compliance and the transient phases are evident. It is clear from the sarcomere length record where to measure the steady-state shortening velocity (phase 4). It is crucial that this velocity is measured at the right time after the quick release; measurements made too early will overestimate the velocity, while measurements made too late will underestimate the velocity.

### Curve Fitting of Force–Velocity Data With Hill’s Equation

In fitting force–velocity data with the hyperbolic Hill equation, equation 1 is usually transformed to an explicit function, that is, shortening velocity as an explicit function of muscle load, as follows:


(2) $$V=c/\left(F+a\right)-b$$

Alternatively, the Hill equation can be expressed as an explicit function of force:


(3) $$F=c/\left(V+b\right)-a$$

**Figure 2:**
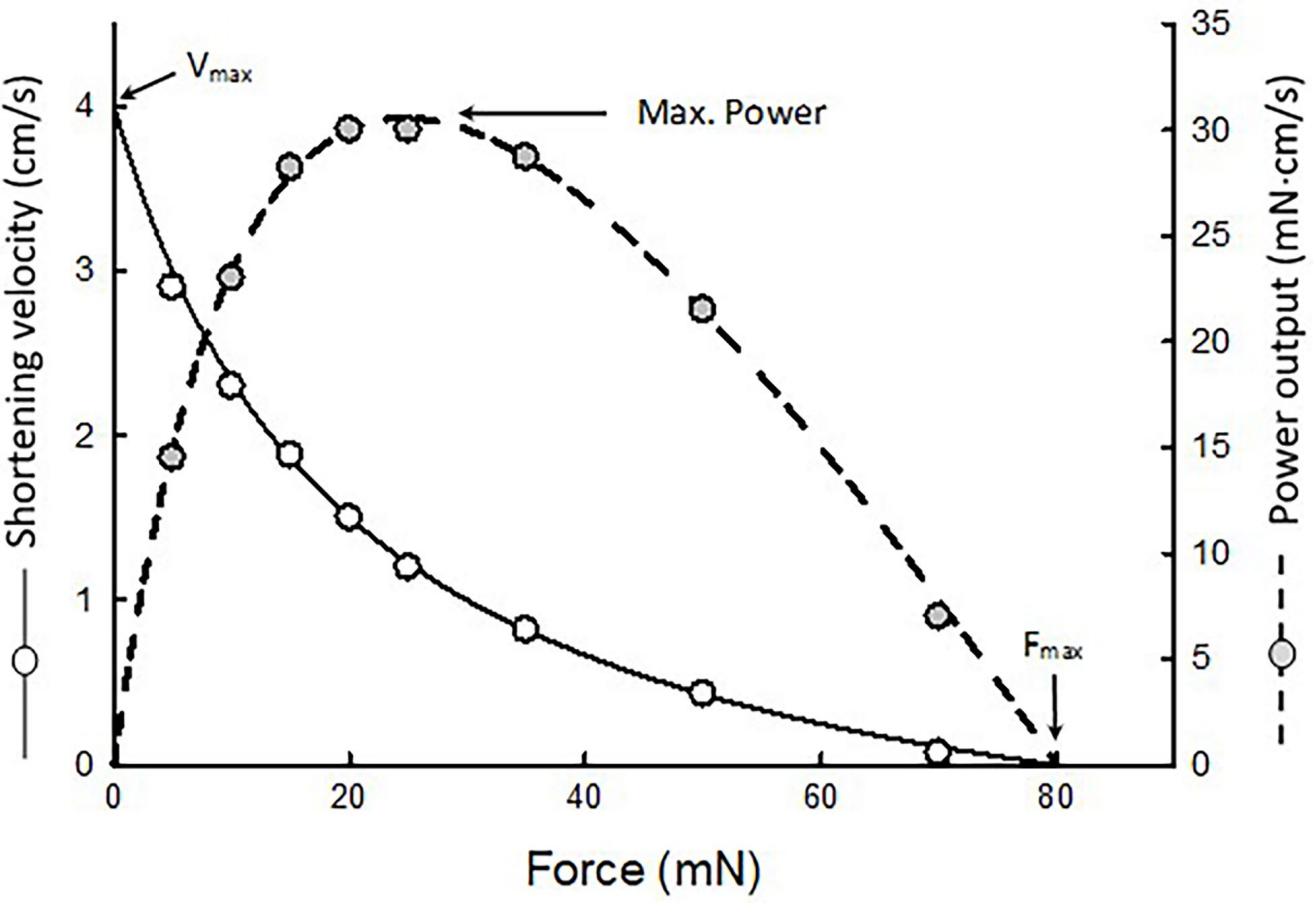
An example of force–velocity data fitted by the Hill equation. The maximal shortening velocity (V_max_) and maximal isometric force (F_max_) are extrapolated from the fitted curve. Because the muscle’s power output is the product of force and velocity, a force–power curve (dotted curve) can be derived from the force–velocity curve.

A non-linear fit of force–velocity data is performed to obtain Hill’s constants *a*, *b*, and *c*. An example is shown in Figure 2. Curve fitting of force–velocity data by the Hill equation usually produces an excellent agreement between the data and the curve, especially in the force range of 5–80% of maximal isometric force (F_max_). Significant deviations of data from the curve can occur in the force ranges of 0–5% F_max_ and 80–100% F_max_. Reasons for the deviation have been discussed in detail by Seow (2013).

Because power equals force times velocity, the muscle’s force–power relationship can be derived from the force–velocity relationship, as shown in Figure 2 (dotted curve). Maximal power typically occurs in the force range of 10–50% F_max_. In this force range, curve fitting of the data is by interpolation. The maximal power output (P_max_) obtained from curve fitting is usually more reliable than the extrapolated values of maximal shortening velocity (V_max_) and F_max_.

### Normalization of Force, Velocity, and Power

The basic contractile unit in striated muscles is the half-sarcomere. In a muscle cell, all sarcomeres have the same structure and function. During contraction, each half-sarcomere contributes equally to the generation of force, shortening, and power by the muscle. Measured mechanical output of a muscle cell can vary depending on the cell size and how the sarcomeres are arranged within the cell. A muscle cell will generate more force if it has more sarcomeres packed in parallel. Because the cell’s cross-sectional area is proportional to the number of sarcomeres *in parallel*, muscle force is normalized by the cross-sectional area of the muscle preparation. Normalized force is called stress, which has the same unit as pressure, for example, kilopascal (kPa) which is also the same as mN/mm^2^.

The number of sarcomeres *in series* is proportional to the muscle length, therefore muscle length or shortening velocity is normalized by the resting length of the muscle preparation, if the sarcomere length is not known. The unit for the normalized shortening velocity is typically muscle length/s. If the sarcomere length is known, then the number of sarcomeres in series can be obtained by dividing the muscle length by the sarcomere length. Of course, the number of half-sarcomeres is twice that of sarcomeres. To normalize shortening velocity, the measured velocity is divided by the number of half-sarcomeres. The unit for normalized shortening velocity is therefore typically μm s^−1^ half-sarcomere^−1^.

The power output of a muscle is proportional to the total number of sarcomeres in the muscle, regardless of their arrangement (i.e., in series or in parallel). The total number of sarcomeres in a muscle is proportional to the volume of the muscle, that is, cross-sectional area of the muscle multiplied by the muscle length. Because muscle volume is more difficult to measure than muscle weight, the latter is typically used to normalize muscle power, since muscle weight is linearly proportional to muscle volume, with the muscle density (~1.06 g/cm^3^) being the constant of proportionality. The unit for normalized power is typically milliwatts per gram of muscle tissue, or mW/g.

The following section further illustrates the rationale for normalization of force–velocity properties.

### Force–Velocity Relationship in Partial Activation and Different Arrangements of Sarcomeres

We have found that in airway smooth muscle, partial activation (defined as a reduction in F_max_, P_max_, and the degree of phosphorylation of the regulatory myosin light chain, but without a change in V_max_) does not result in a change in the curvature of the force–velocity curve (Luo et al., 2019). Mathematically, the curvature of Hill’s hyperbola is determined by the ratio of maximal isometric force over the Hill constant *a*, or the ratio of maximal velocity over the Hill constant *b*, that is:


(4) $$\mathrm{Curvature}=F_{\max}/a=V_{\max}/b$$

**Figure 3:**
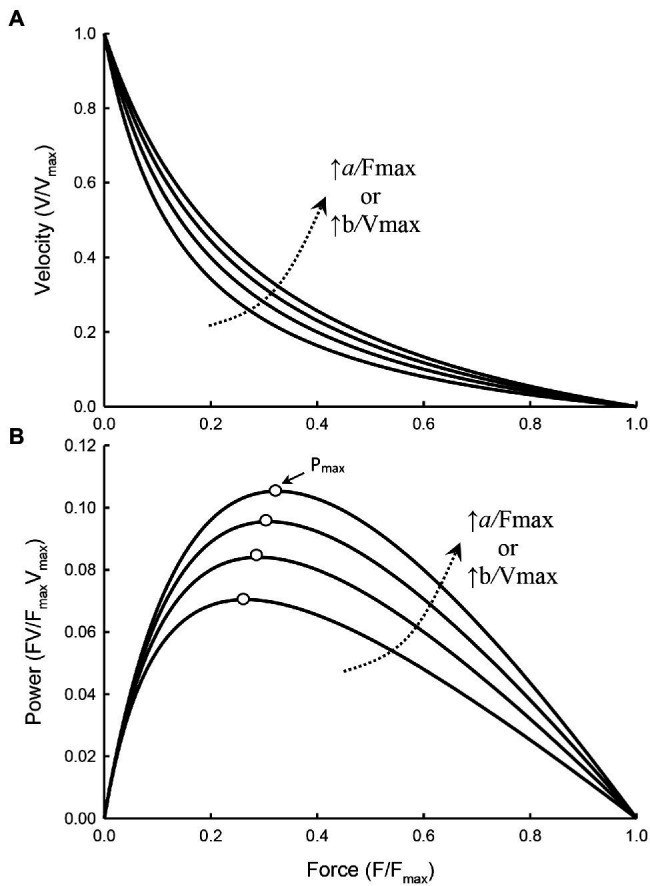
Force–velocity **(A)** and force–power **(B)** curves with the value of *a*/F_max_ and *b*/V_max_ increasing from 0.15, 0.2, 0.25, to 0.3, resulting in a decrease in the curvature of the force–velocity curves (i.e., less curved) and an increase in P_max_ of the force–power curves. The force at which P_max_ occurs (open symbols) also increases as the curvature decreases.

Figure 3 illustrates how curvature of a force–velocity curve could be altered by changes in the ratio of F_max_/*a* or V_max_/*b*.

For interventions that do not alter the kinetics of actomyosin interaction, the force–velocity curvature will not be affected by such interventions. For example, in fully and partially activated muscles where the change in activation is strictly limited to a change in the number of activated crossbridges, with no change in the kinetics of actomyosin interaction, the curvature is not expected to change. The Hill equation for the fully and partially activated muscle can be written as follows:


(5) $$V_{f}=\frac{b\left(F_{\max}-F\right)}{F+a}$$

(6) $$V_{p}=\frac{b_{p}\left(nF_{\max}-F\right)}{F+a_{p}}$$

In equations 5 and 6, *V_f_* and *V_p_* denote shortening velocity of fully and partially activated muscles, respectively, *b_p_* and *a_p_* are Hill’s constants for the partially activated muscle, and *n* is an index of activation with *n* = 1 for full activation and *n* < 1 for partial activation. We have shown that if partial activation only results in a reduction in the number of attached crossbridges, the Hill constant *b* remains unchanged (Luo et al., 2019). That is, *b_p_* = *b*. Because V_max_ is the same in either full or partial activation, and V_max_ occurs when *F* = 0, from equations 5 and 6:


(7) $$V_{\max}=\frac{bF_{\max}}{a}=\frac{bnF_{\max}}{a_{p}}$$

From equation 7 one can derive: *a*_p_ = *na*. The curvature of the force–velocity curve for the partially activated muscle is *n*F_max_/*a*_p_ or *n*F_max_/*na*, or simply F_max_/*a*. The curvatures as defined by equation 4 (F_max_/*a*) for both fully and partially activated muscles are therefore the same.

**Figure 4:**
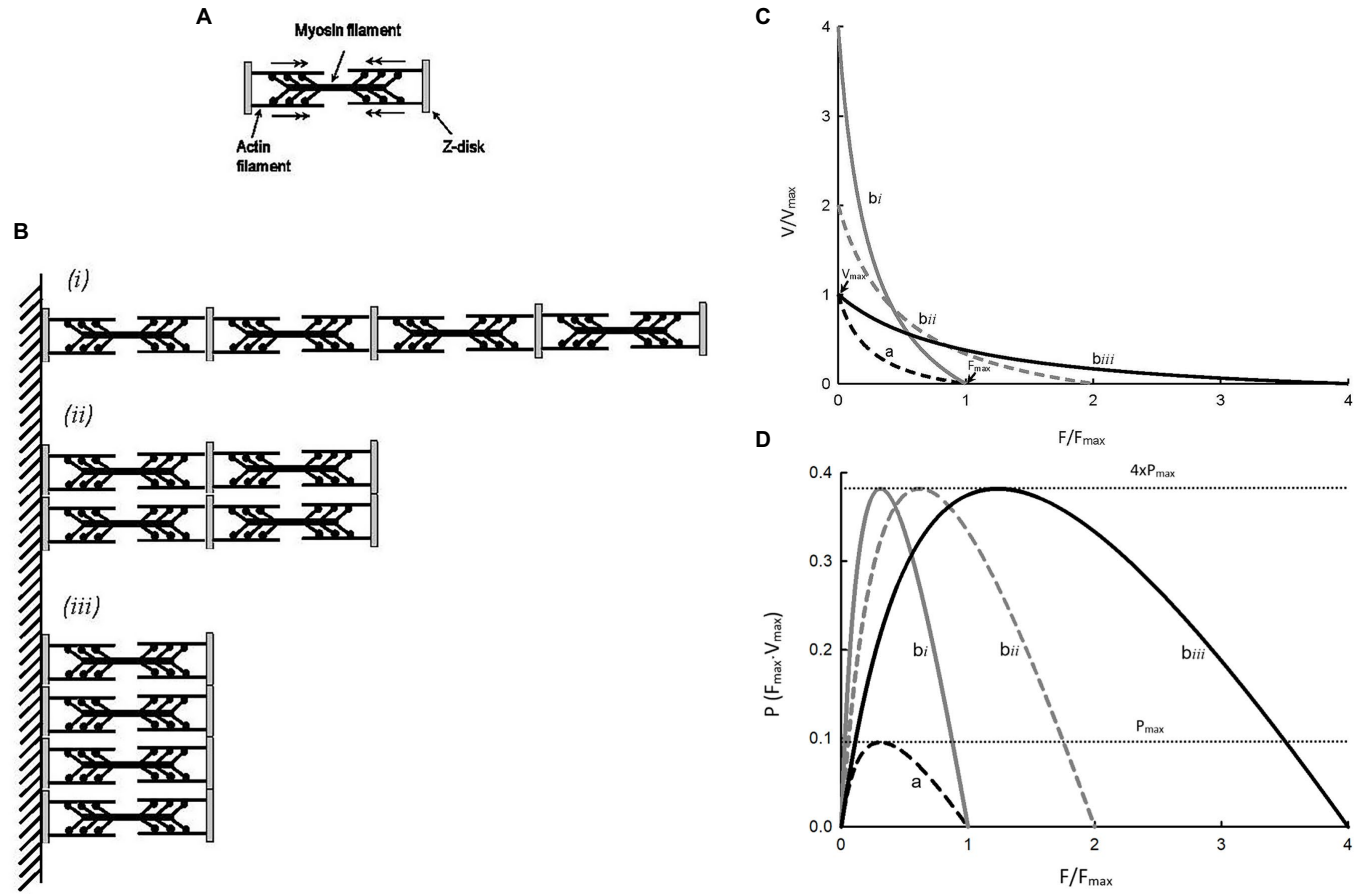
**(A)** Schematic representation of a sarcomere. **(B)** Three different arrangements of 4 identical sarcomeres in parallel and in series. **(C)**Force–velocity curves for a single sarcomere (a) and each of the sarcomere arrangements (**b***i*, **b***ii*, **b***iii*). **(D)** Force–power curves for a single sarcomere (a) and each of the sarcomere arrangements (**b***i*, **b***ii*, **b***iii*).

Hypertrophy or atrophy of a striated muscle cell does not alter the structure or function of individual sarcomeres, but changes their number. Figure 4A shows a simplified illustration of a sarcomere, and Figure 4B shows three different arrangements of four such sarcomeres. Figure 4C shows force–velocity curves for each of the arrangements: **a**, **b***i*, **b***ii*, and **b***iii*. Note that, graphically, curve **b***i* is obtained by scaling the velocity values of curve **a** by a factor of 4; curve **b***ii* is obtained by scaling both force and velocity values of curve **a** by a factor of 2; and curve **b***iii* is obtained by scaling the force value of curve **a** by a factor of 4. Because the crossbridge kinetics is not altered by different geometric arrangements, the shape of the force–velocity curves after normalization by the number of sarcomeres in parallel and in series, **b***i*, **b***ii*, and **b***iii* will all superimpose exactly on curve **a**. In other words, scaling of force and velocity in each of the cases, **b***i*, **b***ii*, and **b***iii*, does not change the curvature of the force–velocity curves. The conservation of curvature can be illustrated mathematically. From equation 7 we learn that scaling the force by a factor *n* will lead to a change in the Hill constant *a* by the same factor. For example, in the case of partial activation, *a*_p_ = *na*. It can be shown that *n* can be any positive number and it does not have to be less than 1 as in the case of partial activation. If we use *a_n_* to denote the Hill constant *a* in a force–velocity relationship where force can be scaled up or down by a factor of *n*, then *a_n_* = *na*. In a similar manner, it can be shown that *b_m_* = *mb*, where *m* is the scaling factor for velocity. An interesting property of the Hill equation is that the constants *a* and *b* are mutually exclusive in that changing the force scale only affects constant *a*, and changing the velocity scale only affects constant *b*. Therefore, for case **b***i* (Figure 4C), *b_m_* = *4b*. The curvature of curve **b***i*, as defined by equation 4 is 4V_max_/*b_m_* or 4V_max_/4*b* or simply V_max_/*b*, is the same curvature as the unscaled curve **a**. For the case **b***ii* in Figure 4C, *a_n_* = 2*a* and *b_m_* = 2*b*. The curvature does not change in this case either, because *n*F_max_/*a_n_* = 2F_max_/2*a* = F_max_/*a* and *m*V_max_/*b_m_* = 2V_max_/2*b* = V_max_/*b*. Finally, for case **b***iii* in Figure 4C, *n*F_max_/*a_n_* = 4F_max_/4*a* = F_max_/*a*. Again, scaling force does not lead to a change in the curvature of the force–velocity curve. It should be pointed out that, in any case, the scaling factor has to be a constant, and not a function.

For the examples shown in Figure 4C, shortening velocity is proportional to the number of sarcomeres in series and force is proportional to the number of sarcomeres in parallel. From Figure 4D, it is clear that power output of a muscle is proportional to the number of sarcomeres, regardless of their arrangements. For curves **b***i*, **b***ii*, and **b***iii*, the maximal power is the same, and it is 4 times the maximal power for curve **a** because they have 4 times more sarcomeres. The examples shown in Figure 4 support the rationale for how force, velocity, and power are normalized, as described earlier in the section *Normalization of force, velocity, and power* above.

### Force–Velocity Relationships With and Without an Internal Load

Force in a force–velocity relationship usually refers to externally applied force or load against which the muscle shortens. The viscous and compressive loads within the muscle tissue are not part of the external load. In the absence of an internal load, the externally applied force is the total force “seen” by the muscle. In most studies, internal loads are neglected because they are relatively small compared to the maximal load (F_max_). However, even with an internal load less than 5% of F_max_, V_max_, and *a*/F_max_ can be significantly altered. Internal loads are ubiquitous in muscle preparations. They stem from any loci within the muscle that offer impediments to shortening. They are present in smooth (Wang et al., 2002; Luo et al., 2019) and cardiac (Chiu et al., 1982; Walley et al., 1991) muscles. The internal load in skeletal muscle is relatively small and may be justifiably neglected, but under some experimental conditions, such as acidosis (Seow and Ford, 1993), the internal load can be significant.

**Figure 5:**
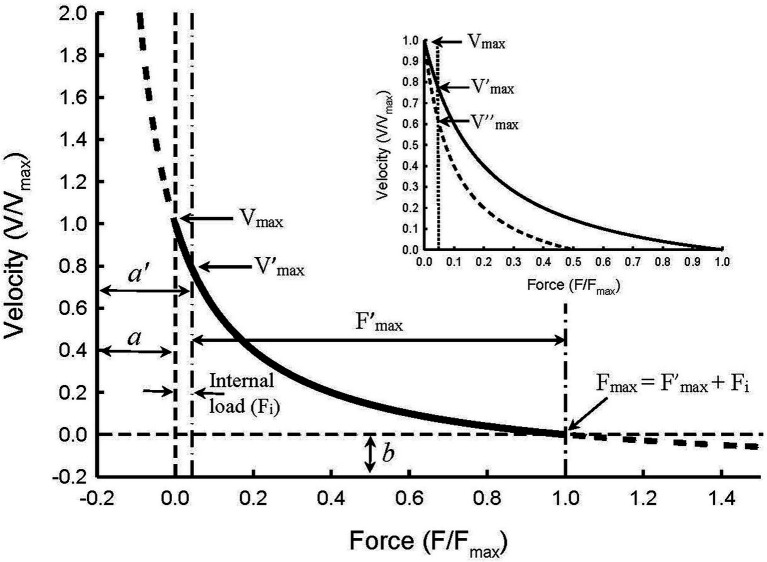
Illustration of force–velocity relationship in the presence of an internal load of 0.05 F_max_ magnitude. The Hill constants *a* and *b* are asymptotes of the hyperbolic curve. An internal load effectively increases constant *a* so that the new asymptote *a*’ equals *a* + F_i_. The internal load also reduces maximal isometric force so that F’_max_ equals F_max_ – F_i_. The apparent force–velocity curvature (F’_max_/*a*’) decreases in the presence of an internal load, compared with the curvature in the absence of the internal load (F_max_/*a*), because F’_max_/*a*’ = (F_max_ – F_i_)/(*a* + F_i_). The maximal velocity seen in the absence of both internal and external loads (V_max_) is not affected by the internal load. However, the internal load causes an apparent decrease in the maximal velocity (from V_max_ to V’_max_). Inset: Illustration of how partial activation in the presence of an internal load causes an apparent decrease in the maximal velocity from V’_max_ at full activation to V″_max_ at half-activation. The changes in V_max_, F_max_, and F_max_/*a* due to an internal load are called “apparent” because the changes are artifacts caused by the internal load and not by changes in the kinetics of actomyosin interaction.

Figure 5 graphically illustrates what an internal load is. Hill’s constants *a* and *b* are the asymptotes of the Hill hyperbola. In a typical force–velocity curve only the portion between V_max_ and F_max_ of the curve is plotted (Figure 5, solid curve). If an internal load is present, the force–velocity curve is truncated and only the portion of the curve between V’_max_ and F’_max_ is plotted. In order for a muscle to shorten, the contractile force developed by the muscle has to overcome both the internal and external loads. An internal load therefore functions to reduce the contractile force of the muscle, similar to partial activation. As in partial activation, an internal load affects the force–velocity relationship by changing the Hill constant *a* but not *b* (Seow and Stephens, 1986; Seow and Ford, 1993; Luo et al., 2019). The curvature of the force–velocity curve within the force range of F’_max_ can be derived from:


(8) $$a\;'/F\;'{}_{\max}=\left(a+F_{i}\right)/\left(F_{\max}-F_{i}\right)$$

where *a*’ is the new asymptote for the curve with an internal load (F_i_), and F’_max_ is the maximal isometric force with an internal load. Because F_i_ is always positive, it follows that *a*’/F’_max_ will always be greater than *a*/F_max_, and the greater the internal load, or ratio of F_i_/F_max_, the greater the value of *a*’/F’_max_ will be. Therefore, even though an internal load does not actually alter the shape of the force–velocity curve (Figure 5), the apparent curvature indicated by F’_max_/*a*’ is decreased.

The inset in Figure 5 illustrates a method with which internal loads can be determined. Without an internal load, V_max_ will not be altered by partial activation of the muscle where only the number of activated crossbridges is reduced without changing the crossbridge kinetics (Luo et al., 2019). Therefore, by extending the force–velocity curve pass the vertical dot-dash line (Figure 5) to where the true zero load is (zero origin of the force axis), all curves with different amounts of internal load will converge to one point on the graph. That point is where both the internal and external loads are zero and where the true maximal shortening velocity (V_max_) occurs. The inset of Figure 5 shows two curves, one for full activation and the other for half-activation. Without taking internal loads into consideration, one would conclude that the maximal velocity obtained under full activation (V’_max_) is greater than that under half-activation (V″_max_). However, if the internal load is considered, we would conclude that the true maximal velocity (V_max_) is not changed, and the apparent difference among V_max_, V’_max_, and V″_max_ is just an artifice created by the internal load and partial activation.

## Hill’s Hyperbola and the Crossbridge Kinetics

Hill’s hyperbola has been broadly used to describe the force–velocity relationship in muscle contraction despite significant deviations of the force–velocity data from the mathematical description at extreme high and low loads [See Seow (2013) for possible explanations for the deviation]. The extrapolated values of F_max_ and V_max_ are therefore approximations. Force–velocity data obtained in the force range between 5–80% F_max_ are much better described by the Hill hyperbola, making the measurement of P_max_ more accurate than the measurements of V_max_ and F_max_. The curvature of the Hill hyperbola (mathematically represented by the term F_max_/*a*) is mainly determined by the force–velocity data obtained within the load range of 5–80% F_max_. Measurement of the curvature is therefore generally reliable. However, without a clear link between the Hill hyperbola and the kinetics of crossbridge cycle, interpretation of the curvature change in terms of physiological mechanisms has been vague.

**Figure 6:**
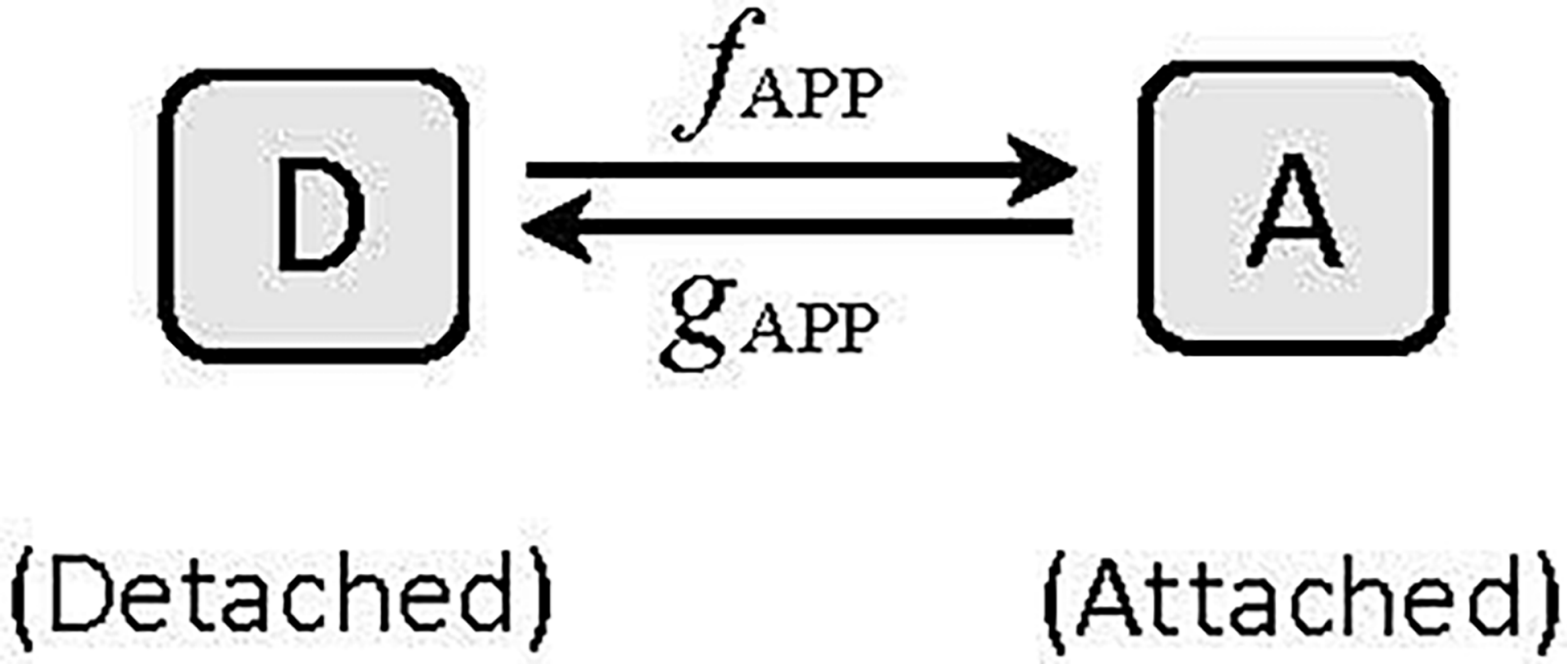
A two-state model of cyclic interaction of myosin crossbridges with actin filaments. A, attached state, D, detached state. *f*_APP_ and *g*_APP_ denote the apparent forward and reverse rate constants, respectively.

When force and velocity in the Hill equation are normalized by their respective maximal values, that is, *F* = F/F_max_, *V* = V/V_max_, the Hill equation (equation 1) can be simplified to:


(9) $$F=\frac{K\left(1-V\right)}{K+V}$$

where *K* is a constant and equals *a*/F_max_ [See Seow (2013) for details of derivation]. The Huxley (1957) model is the simplest example of two-state crossbridge kinetics, consisting of a detached and an attached state (Figure 6). In this model, the actomyosin crossbridges are either in the detached (*D*) or attached (*A*) state, and the fraction of bridges in the detached and attached states sum up to one. That is, *A* + *D* = 1. To link equation 9 to Huxley’s two-state model which assumes that force is proportional to the number of crossbridges in the attached state, *F* becomes *pA*, where *p* is force per bridge and *A* is the number of attached bridges. From the model shown in Figure 6, it can be derived that:


(10) $$A=\frac{f_{APP}}{f_{APP}+g_{APP}}$$

where *f*_APP_ and *g*_APP_ are the apparent attachment and detachment rates, respectively (Seow, 2013). Therefore,


(11) $$F=pA=p\left(\frac{f_{APP}}{f_{APP}+g_{APP}}\right)$$

Because *p* is inversely related to velocity and *g*_APP_ is linearly proportional to velocity (Piazzesi et al., 2007), that is, *p* = 1−*V* and *g*_APP_ = *kV*, (where *k* is a constant of proportionality) equation 11 becomes:


(12) $$F=\left(1-V\right)\left(\frac{f_{APP}}{f_{APP}+kV}\right)$$

By defining that:


(13) $$K=f_{\mathrm{APP}}/k$$

equation 12 becomes *F* = *K*(1−*V*)/(*K* + *V*), which is exactly the same as equation 9, the Hill equation.

In Hill’s equation, *K* = *a*/F_max_, and is inversely proportional to the curvature of the force–velocity curve. We can therefore define the curvature as follows:


(14) $$\mathrm{Curvature}=1/K=F_{\max}/a=k/f_{\mathrm{APP}}$$

Equation 14 provides a crucial link between the curvature of Hill’s hyperbola and the rate of attachment (*f*_APP_) and the velocity-dependent rate of detachment of myosin crossbridges (*k*) in their cyclic interaction with actin filaments.

### Alteration of Force–Velocity Curvature by Changes in Crossbridge Kinetics

After a force–velocity curve has been properly normalized and internal loads taken into account, if its curvature is still different before and after an intervention, one can conclude that the crossbridge kinetics have been altered by the intervention. The intervention can come from Nature during evolution of species where the biochemical properties of isoforms of myosin and actin have been changed. The intervention can also come from changes in the chemical milieu in which actomyosin interaction occurs. The changes in chemical milieu in turn can be initiated by environmental factors, such as temperature and oxygen level, or pathological conditions, such as acidosis and alkalosis. A broader discussion on how interventions modify the force–velocity properties of muscle can be found in a review by Seow (2013). The following discussion focuses on interventions that specifically alter the force–velocity curvature.

### Fast vs. Slow Muscle Types

It has been shown in earlier discussions that changes in the arrangement of sarcomeres, partial activation, and internal loads do not fundamentally alter the force–velocity relationship, because after normalization of force and velocity, the force–velocity curvature remains the same. Fast and slow muscle fibers contain different myosin isoforms. Observations from many studies comparing the force–velocity relationships in fast and slow muscles, revealed that fast muscles, besides having higher V_max_, also have less curvature (lower values for F_max_/*a*; Katz, 1939; Close, 1964; Woledge, 1968; Cecchi et al., 1978; Lännergren, 1978; Luff, 1981; Lännergren et al., 1982; Ranatunga, 1982; Brooks and Faulkner, 1988; Stienen et al., 1988; Barclay et al., 1993; Wahr and Metzger, 1998; Gilliver et al., 2009). This means that fast muscles possess a greater power output than slow muscles do, not only by having a faster shortening velocity, but also by having less curvature in their force–velocity curves. Cardiac myocytes containing the faster isoform α-MHC have a higher shortening velocity and power output than those containing the slower isoform β-MHC. The faster isoform α-MHC is also associated with a reduction in the curvature of the force–velocity curve, F_max_/*a* (Herron et al., 2001).

Because shortening velocity is proportional to the apparent rate of crossbridge detachment (*g*_APP_), and from equation 13, *K* = *f*_APP_/*k*, we can deduce that the molecular mechanisms conferring the phenotype of fast myosin isoforms are (1) a faster rate of crossbridge detachment (*g*_APP_), likely due to an increased rate of dissociation of ADP from its myosin binding site (Siemankowski et al., 1985; Rayment et al., 1993; Elangovan et al., 2012), (2) an increase in the rate of crossbridge attachment (*f*_APP_) and/or (3) a decrease in the velocity–dependent rate of detachment (*k*). The first mechanism gives rise to higher shortening velocities and the second and third mechanisms, individually or together, can explain the increase in *K* value observed in muscles with fast myosin.

It has been observed that the slow tortoise muscle is more energetically efficient than the faster frog muscle and that the former also possesses a greater curvature in its force–velocity curve than the latter (Woledge, 1968). It is possible that a greater value for *k* is associated with the slow tortoise muscle. This makes the dissociation rate of crossbridges from their actin binding sites faster as the shortening velocity increases, reaching its maximal value at V_max_. This could lead to less bridges being dragged into the negative-force region (Huxley, 1957). The improvement in energetic efficiency may come as a result of fewer negatively strained bridges.

### Effects of Temperature

It has been found that V_max_ and *a*/F_max_ both increase with temperature, with a strong correlation between V_max_ and *a*/F_max_ over a temperature range of 10°–35° (Ranatunga, 1984). Furthermore, V_max_ and *a*/F_max_ in fast muscles, compared with those in slow muscles, have higher temperature sensitivity. F_max_ has also been found to be temperature-dependent, and the augmentation of F_max_ by temperature appears to be due to enhancement of a step transition of crossbridges from the detached state to a force-generating state before the release of inorganic phosphate from the myosin head (Ranatunga, 2010). The temperature-dependent increase in V_max_, F_max,_ and *a*/F_max_ will tend to increase the power output of the muscle. Interestingly, analysis by Ranatunga (1998) showed that among the force–velocity parameters, P_max_ has the highest temperature sensitivity. This perhaps can be understood as a consequence of a synergistic effect from the contribution of V_max_, F_max_, and *a*/F_max_ to P_max_. For example, if velocity and force both increase by 10% due to temperature increase, this alone would lead to an increase in power output by 21% because power equals velocity times force, assuming no change in *a*/F_max_. An increase in *a*/F_max_ with temperature will further contribute to the increase in P_max_.

From equation 13 we know that *a*/F_max_ (or *K*) is determined by *f*_APP_ and *k*. A study on the effects of temperature on the crossbridge cycle has revealed that the fraction of detached bridges decreases as temperature increases (Zhao and Kawai, 1994), indicating that *f*_APP_ is enhanced and/or *k* is diminished by increasing temperature. In homeothermic species, the temperature sensitivity of *a*/F_max_ is perhaps physiologically less relevant. For ectotherms, such as fish living in water with large, depth-dependent temperature gradient, a large change in *a*/F_max_ (hence P_max_) could significantly impact their function. This is perhaps why, in some cold-water fish, *a*/F_max_ is temperature insensitive (Johnston and Salamonski, 1984; Johnston and Sidell, 1984). In carp red muscle, the temperature-dependent change in *a*/F_max_ is opposite to that in mammalian skeletal muscle (Rome and Sosnicki, 1990). This is perhaps a compensatory mechanism for ectotherms to reduce power loss in muscle at low temperature by increasing *a*/F_max_ (decreasing the curvature).

### Effects of Metabolites From ATP Hydrolysis

In muscle contraction, the immediate energy source is ATP hydrolysis. When an ATP molecule is hydrolyzed, P_i_ (inorganic phosphate), ADP (adenosine diphosphate), and H^+^ (hydrogen ion) are produced. The hydrolysis products are known to alter the kinetics of the crossbridge cycle and hence the force–velocity relationship.

In experiments using membrane-permeabilized muscle fibers, it has been shown that when ADP release is partially blocked by high concentrations of ADP in the intracellular milieu, F_max_ is increased and V_max_ is decreased (Cooke and Pate, 1985; Seow and Ford, 1997). Furthermore, the curvature of the force–velocity curve is reduced by high concentrations of ADP (Seow and Ford, 1997). The increase in *a*/F_max_ (reduced curvature) is likely due to a decrease in the sensitivity of *g*_APP_ on shortening velocity, that is, a reduction in the value of *k* in equation 13. The effect of slowing ADP release in the crossbridge cycle can also be demonstrated in experiments using a bipyridine compound (amrinone), which is known to increase ADP affinity to myosin. The presence of amrinone enhances F_max_, diminishes V_max_, and augments *a*/F_max_ (Albet-Torres et al., 2009). Low [ATP] has a very similar effect as high [ADP] on the crossbridge cycle and hence the force–velocity relationship (Ferenczi et al., 1984; Cooke and Pate, 1985; Stienen et al., 1988; Seow and Ford, 1997). Furthermore, low [ATP] has been found to cause an increase in *a*/F_max_ (Ferenczi et al., 1984; Stienen et al., 1988; Seow and Ford, 1997; Wakayama and Yamada, 2000; Cheng et al., 2020).

The effect of high [H^+^] on force–velocity curvature is more complicated, because it affects multiple points in the crossbridge cycle. Detention of crossbridges in the low-force states by high (H^+^; Seow and Ford, 1993) tends to decrease *f*_APP_, which would increase the curvature (Overgaard et al., 2010). On the other hand, slowing of ADP release (Debold et al., 2008) would tend to decrease the value of *k* and hence curvature (Albet-Torres et al., 2009). This may explain the mixed reports on the effects of high [H^+^] on the force–velocity curvature. In addition, the high [H^+^] effect on the force–velocity curvature is dependent on temperature and muscle type. For example, at low temperature (15°C), Knuth et al. (2006) found that in type I muscle, *a*/F_max_ increased at low pH and decreased at high pH, but at a higher temperature (30°C), *a*/F_max_ decreased at low pH and increased at high pH. In type II muscle at 15°C, *a*/F_max_ increased at low pH and decreased at high pH, just like that in type I muscle, but at 30°C, pH had no significant effect on *a*/F_max_ in type II muscle. In rabbit psoas (fast) muscle at 10°C, Cooke et al. (1988) found no significant change in *a*/F_max_ due to pH change. For the same muscle at 1.5°C, Seow and Ford (1993) found a significant increase in *a*/F_max_ at low pH. From the scattered results one can conclude that to understand the effect of low pH on physiological function of muscle, the force–velocity parameters should be measured at or near body temperature.

### Effects of Muscle Fatigue

At the muscle cell level, fatigue is often a consequence of energy demand exceeding supply. The immediate energy supply for muscle contraction is derived from the intracellular pool of ATP, normally at a concentration of about 5 mM. Metabolism of ATP produces metabolites that include ADP, P_i_, and H^+^. The intracellular concentration of ATP is usually maintained at a constant level by buffering systems that involve phosphocreatine, glycolysis, and oxidative phosphorylation. When oxygen supply lags demand, lactic acid accumulates, leading to acidosis. To make things worse, both P_i_ and H^+^ reduce myofibrillar Ca^2+^ sensitivity (Debold et al., 2006; Nelson and Fitts, 2014). Effects of muscle fatigue on the force–velocity relationship are therefore multifaceted due to the many contributing factors.

The consequence of muscle fatigue involves a reduction in the ability of the muscle to generate force and reduced shortening velocity. However, there are mixed reports regarding changes in the curvature of the force–velocity curve (Barclay, 1996; Fitts, 2008; Jones, 2010; Devrome and MacIntosh, 2018; Kristensen et al., 2019, 2020). The ratio of ADP/ATP is usually well maintained and is thought to play little role in causing fatigue (Cooke, 2007). Reduction in myofibrillar Ca^2+^ sensitivity leads to a reduced number of activated crossbridges, similar to the situation in partial activation. If lower myofibrillar Ca^2+^ sensitivity is equivalent to partial activation (discussed above), it would decrease F_max_ but have no effect on *a*/F_max_, as reported by Langeron et al. (1999). However, other studies showed that lowering intracellular [Ca^2+^] led to a slight increase in *a*/F_max_, and augmenting intracellular [Ca^2+^] led to a decrease in *a*/F_max_ (Kristensen et al., 2018). The effect of intracellular [Ca^2+^] on the force–velocity curvature is therefore inconclusive. Note that the presence of an internal load tends to increase the ratio *a*/F_max_, as discussed above. Taking internal loads into account in the determination of force–velocity properties may help to resolve some of the discrepancies.

In a muscle cell, ADP produced from ATP hydrolysis is used in the phosphocreatine buffering system to replenish ATP, but P_i_ produced by ATP hydrolysis is not used in this buffering system and therefore can accumulate inside the muscle cell. As discussed above, high [P_i_] depresses F_max_ but has little effect on V_max_. In mild fatigue, a small decrease in F_max_ occurs without a significant decrease in V_max_ (Edman and Mattiazzi, 1981; Jones et al., 2006). It is likely that P_i_ accumulation is responsible for the initial phase of muscle fatigue (Wilson et al., 1988). In later phases of fatigue, accumulation of H^+^ and lactic acid, combined with high [P_i_] likely lead to a large depression of F_max_ and V_max_ due to the direct effects of low pH and high [P_i_], and indirect effect of low myofibrillar Ca^2+^ sensitivity. Low pH at near body temperature has been shown to increase the curvature (decrease in *a*/F_max_; Knuth et al., 2006). Low pH is therefore one of the contributors to the increase in force–velocity curvature observed in muscle fatigue. As discussed above, high [H^+^] detains crossbridges in the low-force states and reduces the apparent rate of attachment (*f*_APP_). A similar conclusion is reached by Jones (2010) in that there must be a substantial decrease in the rate constant for attachment in the Huxley (1957) model to account for the observed decrease in power and increase in force–velocity curvature in fatigued muscle.

## Conclusion

Mechanical manifestation of muscle activation captured in the force–velocity relationship is a window through which the molecular events of the crossbridge cycle can be observed. The presence of an internal load can lead to an apparent decrease in the curvature of a force–velocity curve, and the apparent decrease in the curvature can be exaggerated under conditions of partial activation or interventions that change the number of activated crossbridges. All these interventions do not alter the true curvature of the force–velocity curve, because, after proper normalization, the apparent changes in the curvature will disappear. When the kinetics of the crossbridge cycle is interrupted by experimental interventions, changes in the true curvature of the force–velocity curve can be linked to specific changes in the velocity-dependent rate functions governing the cyclic actomyosin interaction.

## Author Contributions

KS and CS contributed to the drafting and editing of the review. All authors contributed to the article and approved the submitted version.

## Funding

This work was supported by a Discovery Grant from the Natural Science and Engineering Research Council (NSERC) of Canada.

## Conflict of Interest

The authors declare that the research was conducted in the absence of any commercial or financial relationships that could be construed as a potential conflict of interest.

## Publisher’s Note

All claims expressed in this article are solely those of the authors and do not necessarily represent those of their affiliated organizations, or those of the publisher, the editors and the reviewers. Any product that may be evaluated in this article, or claim that may be made by its manufacturer, is not guaranteed or endorsed by the publisher.
